# Association of Secondary Primary Malignancies in Cutaneous Lymphoma: A Narrative Review

**DOI:** 10.3390/diagnostics15243150

**Published:** 2025-12-11

**Authors:** Yu-Hsiang Hung, Pa-Fan Hsiao

**Affiliations:** 1 Department of Dermatology, MacKay Memorial Hospital, Taipei 10449, Taiwan; xdkururu@gmail.com; 2 Department of Medicine, MacKay Medical University, New Taipei City 25245, Taiwan; 3 Department of Cosmetic Applications and Management, MacKay Medicine, Nursing and Management College, Taipei 11260, Taiwan

**Keywords:** cutaneous lymphoma, cutaneous T-cell lymphoma, cutaneous B-cell lymphoma, second primary malignancy, epidemiology, risk factors, prognosis

## Abstract

Cutaneous lymphomas are a heterogeneous group of extranodal non-Hodgkin lymphomas with distinct clinical and biological features, broadly classified into cutaneous T-cell lymphomas (CTCL) and cutaneous B-cell lymphomas (CBCL). With improved survival due to early detection and therapeutic advances, the emergence of second primary malignancies (SPMs) has become a clinical concern. SPMs, defined as new, distinct malignant neoplasms arising synchronously or metachronously with the index cancer, can significantly impair prognosis and quality of life. In this narrative review, we meticulously examine the current literature, to synthesize evidence on SPMs’ incidence and risk factors in patients with primary cutaneous lymphomas. Evidence from population-based and institutional studies consistently demonstrates elevated risks of hematologic and solid tumors in CTCL. By contrast, data on CBCL remain limited, though recent population-based analyses suggest increased risks of certain hematologic malignancies and solid tumors. We further propose development mechanisms for SPMs, including treatment-related mutagenesis, shared genetic susceptibilities, chronic antigenic stimulation, and immune dysregulation. Lastly, we highlight the clinical implications of these findings, underscoring the need for vigilant surveillance, patient education, and tailored screening strategies. Future research should prioritize large-scale, prospective, and molecularly integrated studies to refine risk stratification and guide personalized survivorship care of this vulnerable population.

## 1. Introduction

Cutaneous lymphomas represent a heterogeneous group of extranodal non-Hodgkin lymphomas (NHL) that arise from the malignant proliferation of immune cells with a particular affinity for the skin. Primary cutaneous lymphomas (PCLs) are defined as lymphomas characterized by confinement to the skin at the time of diagnosis, regardless of subsequent extracutaneous site involvements during follow-up. Conversely, secondary cutaneous lymphomas (SCLs) refer to systemic lymphomas or leukemias that further invade the skin, representing a manifestation of disseminated diseases associated with a worse prognosis than PCL [1,2,3]. As the histopathology of PCLs and SCLs can appear similar or even identical, careful staging investigations are essential to accurately establish the diagnosis.

Within the spectrum of PCLs, two broad categories predominate: cutaneous T-cell lymphomas (CTCL) and cutaneous B-cell lymphomas (CBCL). CTCLs include mycosis fungoides (MF) and Sézary syndrome (SS) as the most common subtypes; CBCLs, by contrast, encompass entities such as primary cutaneous marginal zone lymphoma (PCMZL), primary cutaneous follicle center lymphoma (PCFCL), and primary cutaneous diffuse large B-cell lymphoma, leg type (PCDLBCL, LT) [1]. Each of these subtypes displays distinct clinical presentation, histopathological features, and prognostic implications. Immunohistochemical profile of cutaneous lymphomas and their differential diagnoses are provided in Supplementary Table S1 [4,5,6].

As survival of patients with cutaneous lymphomas improves due to earlier detection and more effective therapies, extended life expectancy inherently increases the cumulative risk of second primary malignancies (SPMs) over time. SPMs are broadly defined as new, distinct malignant neoplasms that arise independently of the index cancer, a cancer diagnosed in a patient without past history of malignancy, and they may occur synchronously (both tumors are diagnosed within a specific time period) or metachronously (the second cancer diagnosed after the index cancer) [7]. It is worth noting that the definition of cut point between “synchronous” and “meta-chronous” differs across studies.

The development of an SPM can adversely affect prognosis and impair quality of life [8]. For instance, the cumulative mortality from SPM was found to exceed that from Hodgkin lymphoma 15 years after diagnosis [9]. One SEER-based study also revealed elevated risk of death from SPMs in individuals with MF [10]. Such findings had transformed SPMs from an incidental observation into a meaningful survivorship issue. Thus, understanding the incidence and types of SPMs is crucial. Moreover, delineating the biological underpinnings of SPM development may provide insights into shared genetic susceptibilities and molecular pathways, which also improve the survivorship care.

Despite growing recognition of this problem, research into SPMs in cutaneous lymphomas was limited. Over the past three decades, the majority of studies have focused on CTCL, with multiple registry-based and institutional reports identifying increased risks of cutaneous malignancies, solid tumors, and hematologic neoplasms in this population [11,12,13,14,15,16,17,18,19,20,21,22,23,24,25,26,27,28,29,30,31,32,33,34,35,36,37,38,39,40,41,42]. By contrast, the relationship between CBCL and SPMs remains far less defined. Most of the studies involved institution-based retrospective cohorts [39,43,44,45], often with small sample sizes, or published as brief communications or letters. Although few population-based studies have suggested links between CBCL and certain secondary malignancies [46,47,48], the strength of evidence is weak, and mechanistic explanations remain largely speculative.

Given these gaps, a comprehensive review of the available evidence is warranted. The purpose of this article is thus to summarize the current knowledge regarding SPMs in cutaneous lymphomas, and hopefully stimulate future research effort focused on risk assessment, monitoring strategies, and detailed underlying mechanisms in such patients.

## 2. Incidence of SPMs in CTCL

As MF and SS constitute two-thirds of primary CTCL [6], early studies focusing on MF have shown an increased risk of both hematologic malignancies, including Hodgkin lymphoma (HL) and NHL, as well as solid tumors, such as lung, renal, breast, colon, and pancreatic cancers, and melanoma, secondary to MF [12,13,14,17,19,25,26,27,28,40]. In most of these studies, the standardized incidence ratio (SIR) was employed to assess relative risk. SIR is typically defined as the ratio of the observed SPMs in the study population to that of the expected malignancies in the general population after demographic factors are adjusted. An SIR of one signifies that the incidence is equivalent to that of the general population. Three retrospective cohort studies utilizing Surveillance, Epidemiology, and End Results (SEER) data between January 1973 and December 2015 reported different SPMs in their respective studies [11,12,13]. Although the earliest SEER-data-based study stated that an increased risk of lung and colon cancers and NHL was observed in 544 patients with MF or SS between 1973 and 1983 [11], Huang et al. found no increased incidence of colon and lung cancers in their updated SEER cohort study ranging from 1984 to 2001. After aggregating both study periods from 1973 to 2001, an elevated incidence of lung cancer, HL, NHL, and overall malignancy was observed [12]. Furthermore, the latest SEER-data-based study, including 6742 cases between 2000 and 2015, reported elevated SIRs in both hematologic malignancies and solid tumors, including lung, melanoma, breast, prostate, renal, and colon cancers [13]. In contrast to the two previous studies that excluded patients developing SPM within the first 2 months, Goyal et al. extended the latency exclusion period to 12 months owing to an observed surge in SIR shortly after MF and SS were diagnosed [13]. This surge may be attributed to the initial cancer surveillance and work-up. Non-Hispanic black patients were diagnosed with MF and SPM at a younger age than the other ethnic groups [13]. In another retrospective, single-center study including 672 patients with CTCL, non-White patients tended to develop SPM with shorter latency, although the result was not significant [18]. Given the discrepancy between these studies, the need for more rigorous screening of solid tumors in patients with MF and SS remains debatable. Studies concerning MF and SS are summarized in Table 1.

The second most common CTCL subgroup is the primary cutaneous CD30-positive T-cell lymphoproliferative disorder. Both lymphomatoid papulosis (LyP) and primary cutaneous anaplastic large cell lymphoma (pc-ALCL) fall under this category. Although LyP manifests as an indolent, self-limiting, and recurrent cutaneous lymphoma with an excellent prognosis [1,49], some studies have found an association between LyP and other hematological malignancies (that is, all lymphomas diagnosed before, concomitant, after the LyP were included), varying from 13.8 to 51.7% [29,30,31,32,34,35,36,37]. The most commonly associated malignancy is MF, followed by anaplastic large-cell lymphoma [29,30,31,32,34,36,37]. Risk factors include male sex [32,36,37], old age at diagnosis [30,34,37], type B and C LyP [32,34], and positive T-cell receptor gene rearrangements [31]. Although some authors have suggested that a high rate of association was observed due to prolonged follow-up (5–30 years) and was supported by the cumulative risk of progression [30,38], the recent largest retrospective study conducted in the Netherlands found no significant difference between patients followed up for a median of 217 months and the whole study group [29]. This finding was also substantiated by the Kaplan–Meier curve depicting the development of lymphoma plotted against time, which stabilized at approximately 20% [29]. Similarly, some studies have indicated a higher incidence of non-hematologic malignancies in patients with LyP. Cutaneous squamous cell carcinoma (SCC), malignant melanoma, and bladder cancer have also been documented [29,32].

In contrast to LyP, very few studies have investigated the risk of SPM in patients with pc-ALCL. A SEER-based study that included pc-ALCL from 1992 to 2011 reported elevated SIR of HL, NHL, and urinary cancers following pc-ALCL diagnosis [22]. Joshi et al. further expanded the inclusion period from 1973 to 2020. The SEER were also consistent with those of a previous study, except that melanoma and respiratory tract malignancies were included [33]. Studies concerning LyP and ALCL are summarized in Table 2.

## 3. Risk Factors for SPM in CTCL

### 3.1. Age

Based on SEER data from 1992 to 2011 [22], age significantly influences the risk of SPMs in CTCL patients. For all SPMs, SIR significantly elevated in patients older than 40 years old. Patients aged 20~39 exhibit the highest risk for secondary NHL (SIR, 12.65 [95% CI, 1.53–45.69]), while those aged more than 60 show the highest risk for HL (SIR, 11.17 [95% CI, 1.35–40.36]). No SPMs were observed in pediatric patients (0~19 years old), probably owing to low case numbers (20 patients).

For patients with MF, the largest SEER-based study involving 6742 patients reported significantly elevated SIR for all SPMs in patients older than 30 years old after stratified by age at diagnosis of MF [13]. Although SIRs declined with increasing age, they all remained statistically significant. In the subset analysis, those aged between 30 and 50 years old faced substantially higher risk than older patients, particularly for solid tumors such as lung, breast, and prostate cancers. A similar trend was also observed for nodal and extranodal NHL. In MF & SS patients under 30 years old, a population study analyzing SEER data base and California Cancer Registry both reported increased risks of all SPM (SIR 3.40 and 3.45, respectively) [15]. Still, the risk is only statistically significant based on SEER database, with lymphoma and melanoma being the most frequently diagnosed.

For LyP, only institution-based studies suggest age as a probable risk factor. By multivariate analysis, Cordel et al. and Baykal et al. both demonstrated that older age was associated with second lymphoma development (OR, 1.05 per year [95% CI, 1.01–1.08]; *p* = 0.011; OR, 1.05 [95% CI, 1.01–1.08]; *p* = 0.03, respectively) [30,34]. For non-lymphoid malignancies, an earlier study observed that LyP patients who developed SPMs were significantly older at study entry (66 vs. 43 years, *p* = 0.002) [35].

### 3.2. Gender

Some population-based cohorts suggest that being female confers a disproportionate susceptibility to SPM following CTCL, especially in MF and SS [13,14,22]. The SEER-based cohort analyzing all CTCL found that SIR of overall SPM was higher in female (SIR, 1.57 [95% CI, 1.22–1.98]) [22]. However, men were particularly predisposed to HL (SIR, 8.4 [95% CI, 1.02–30.35]), whereas women demonstrated increased risk of bronchopulmonary malignancies (SIR, 2.4 [95% CI, 1.37–3.89]). Two subsequent SEER-based studies narrowed the analysis to MF (and SS). Almukhtar et al. reported a higher SIR in women (SIR, 1.42 [95% CI, 1.23–1.63]) than in men (SIR, 1.11 [95% CI, 0.98–1.25]) after stratified by gender [14]. Female patients showed significantly increased incidence of lung cancer, chronic lymphocytic leukemia, HL, and NHL, while men demonstrated excess risk for HL and NHL. Goyal et al. confirmed this pattern, with women again exhibiting higher overall risk (SIR, 13.95 [95% CI, 12.09–16.02]; *p* < 0.033) and were younger at SPM diagnosis (mean, 61.9 years vs. 64.3; *p* < 0.05) [13].

On the contrary, being male has been associated with an elevated risk of SPM in LyP. One retrospective cohort showed that men with LyP had a nearly threefold increased likelihood of developing associated lymphomas compared to women (*p* = 0.001) [32]. Likewise, a multicenter study from Canada that included 70 patients showed similar findings (OR, 3.16 [95% CI, 1.16–8.59]; *p* = 0.03) [37].

### 3.3. Stage of MF

With regard to the stage of MF, studies have shown different results. In a single-center retrospective cohort study from Turkey, advanced stage (stage IV) was significantly associated with secondary solid tumor (OR, 21.96 [95% CI, 2.04–839.66]) [25]. Another single-center study from Minnesota similarly found that tumor-stage MF had a markedly higher risk compared with patch/plaque stage MF (HR, 4.2 [95% CI, 1.27–12.5]; *p* = 0.018) in multivariate analysis [26]. The most recent and largest SEER-based analysis found elevated SIR for SPM across all MF stages. However, there was no significant difference between the early (IA–IIA) and late stages (IIB–IVB). A potential explanation is that the staging information was only available for approximately 45% of patients included in the dataset. The incomplete data limits the reliability of comparisons between different disease stages. Moreover, the study utilized the most recently documented stage for each patient, which did not account for those who may have progressed to a more advanced stage over time [13].

### 3.4. Histologic Subtype of LyP

The role of histologic subtypes in predicting SPM among patients with LyP remains debated. Two studies provide evidence supporting type B as a significant risk factor. In a Canadian cohort, patients with type B LyP had markedly increased odds of associated hematologic malignancies (OR, 5.14 [95% CI, 1.27–3.26]; *p* = 0.024) [37]. Similarly, in a larger series of 180 patients, both type B and type C were predictive for developing lymphoma, while type D appeared protective [32]. In contrast, other investigations with smaller sample sizes did not confirm this association [30,35].

## 4. Incidence of SPMs in CBCL

In 2017, Chan et al. first reported a high rate of SPM in CBCL [44]. They included 51 patients with primary CBCL from their cutaneous lymphoma database in the United Kingdom who were followed up for a median time of 36 months. Ultimately, 13 cases were noted (13 of 51 patients, 25.5%); however, most had other cutaneous malignancies (six BCC and two SCC). Only one case of solid tumor (breast cancer) was recorded. For hematologic malignancies, five cases were identified (two systemic NHL, two HL, and one chronic lymphocytic leukemia). Some patients developed >1 SPM during follow-up. The median and mean latency times were unknown. Another single-institution retrospective study with a smaller sample size conducted in Spain drew similar conclusions [45]. They identified six SPMs in 16 primary CBCL (16.67%), all of which were solid tumors. Gastrointestinal cancer was the most commonly identified. The mean SPM onset time was 0.43 years. Subsequently, two tertiary referral centers in Italy and Spain conducted larger studies [43]. They further defined SPM as metachronous (diagnosed ≥ 6 months after primary CBCL), synchronous (diagnosed ≤ 6 months before and after primary CBCL), and previous (diagnosed ≥ 6 months before primary CBCL) cases. Overall, 40 patients with ≥1 SPM were noted among 144 patients (27.8%); 27 cases were other cutaneous malignancies, most of which were BCC; 13 patients had second primary solid tumors, with prostate cancers being the most common (4 cases); and only two hematologic malignancies were found (chronic myelomonocytic leukemia and HL). When considering both metachronous and synchronous diagnoses, the mean and median SPM onset times were 71.5 and 48 months, respectively (1–264 months). PCMZL was the most common SPM subtype.

Although most studies were performed in Western countries, Kim et al. conducted the only retrospective study to date focusing on Asian patients [39]. They included 98 patients with CBCL (55 primary and 43 secondary CBCL) from Asan Medical Center between 1997 and 2016 and defined metachronous primary cancers as those diagnosed ≥2 months after cutaneous lymphoma diagnosis. In summary, the most common prechronous and synchronous primary malignancies (diagnosed within 2 months before cutaneous lymphoma diagnosis) were gastrointestinal (n = 3), prostate (n = 3), and lung (n = 2) cancers; however, only four metachronous SPMs (colon, liver, lung, and skin cancers) were identified. The average latency period between diagnoses was 24.0 ± 21.9 months. Notably, these numbers summed up SPM from both primary and secondary CBCL; however, we could not discriminate them, respectively, based on the context.

In the past few years, two SEER-based studies provided evidence of higher SIR of SPM in primary CBCL [46,47]. In the study by Banner et al., patients with CBCL had a 54% higher risk of SPM than the general population. The risk factors for SPM were sex-dependent and increased within 1 year of primary CBCL diagnosis. Women had higher risks of thyroid, renal, and lung cancers, whereas men had increased risks of cutaneous melanoma, bladder cancer, and prostate cancer [47]. The short latency of the SPM from the CBCL may result from the initial workup and frequent healthcare interactions. Soon after, Shah et al. using the same database within the same period but explicitly restricted the inclusion criteria. Not only did they filter for primary CBCL confirmed by histopathological analysis, but they also limited the CBCL to the three most common subtypes, namely PCMZL, PCFCL, and PCDLBCL. SPMs diagnosed within one year of primary CBCL diagnosis were excluded because temporary surge in SIR directly after primary cancer were observed. They eventually included 3757 patients and reported elevated SIR for all SPMs (SIR, 1.37 [95% CI, 1.23–1.52]), especially hematologic malignancy, prostate cancer, and cutaneous malignancy (excluding BCC and SCC). The authors further identified increased risk in patients with white race, 50~74 years old, and early-stage lymphoma. Compared to men, women had shorter latency to SPM (9.9 vs. 12.2 years) [48]. Selective studies concerning primary CBCL and SPMs are summarized in Table 3.

## 5. Hypothesis for Elevated Risk of SPM in Patients with Cutaneous Lymphoma

SPM development in patients with cutaneous lymphomas is a multifactorial process involving immunological, genetic, iatrogenic, and environmental factors. Moreover, most studies focused on CTCL, while articles explaining that in CBCL remain sparse.

### 5.1. CTCL

For CTCL, some authors suggested that ultraviolet (UV) radiation, including both UVB and therapeutic PUVA (psoralen with UVA), can induce cutaneous immuno-suppression and DNA damage [26,28,50]. UVA induces indirect oxidative stress and free radicals, whereas UVB directly induces pyrimidine dimers, both of which can initiate carcinogenesis. Although PUVA therapy increases the risk of SCC and, more rarely, BCC, its direct association with non-cutaneous malignancies remains unclear, especially in patients receiving < 150 treatments [26,51]. Similarly, radiation therapy may also promote oncogenesis, although reported cases remain rare [28,52]. Furthermore, cytotoxic chemotherapy may impair systemic immune function and DNA integrity, potentially contributing to secondary cancer development [22,27,39,44].

Some researchers prefer chronic inflammation and immune dysfunction to iatrogenic influences as a more reasonable explanation, as no consistent correlation was observed between treatment modalities and SPM risk [26]. Persistent activation of immune cells and stromal cells will collaboratively create a protumorigenic and immunosuppressive milieu [53], which can sometimes be portrayed by immunohistochemical (IHC) studies [54]. For instance, using anti-tryptase antibodies, increased mast cell density was found within lymphoma infiltrates and at the periphery [55], and mast cell-derived mediators such as histamine, tryptase, IL-10, and TGF-β can contribute to extracellular matrix remodeling, angiogenesis, and immunosuppression [56,57,58]; single-cell and spatial transcriptomic analyses in MF have identified inflammatory cancer-associated fibroblasts (CAF) within the tumor microenvironment [59], and CAF-stromal cell interactions are also central to tumor development [60]; reduced Ku70 protein expression was shown in CTCL [61], and these impairments are also found in breast cancer [62,63]. Furthermore, a reduced number of interferon-γ-secreting lymphocytes can resemble the immunosuppressive profile observed in acquired immunodeficiency syndrome [64,65]. These immunological alterations are compounded by impairments in DNA repair mechanisms and prolonged repair time in lymphocytes [66]. Genetic factors may influence the risks of developing SPM, as CTCL and SPM sometimes shared the same gene mutation. Previous report had recorded *FAT1* mutation in MF [67] and other solid tumors [68]. Last but not least, viral infection and environmental exposure have also been hypothesized, which can be involved in carcinogenesis [69].

### 5.2. CBCL

The mechanisms underlying increased SPMs in the CBCL remain poorly understood. Currently, evidence to assert that the same immune-mediated pathways operate in CBCL and CTCL is limited. As we integrate current reports, the following explanations may be offered for this intricate issue. First, recent advances in genomic profiling have revealed significant genetic alterations in primary CBCL, suggesting that genetic susceptibility may play a role not only in lymphomagenesis but also in the predisposition to SPMs [70]. Although the direct connection between these mutations and non-hematologic SPMs remains unclear, the recurrent involvement of oncogenic signaling pathways and immune evasion genes implies plausible correlations [70,71]. For instance, PCDLBCL-LT, frequently harbors activating mutations in *MYD88* and *CD79B*, both of which are integral to B-cell receptor signaling and NF-κB activation [71,72,73,74]. These mutations contribute to prolonged B-cell survival and increased genomic instability, potentially creating an environment conducive to secondary oncogenesis. Mutations in oncogenes or tumor suppressors in other oncogenic pathways (*STAT3*, *pIK3R1*, and *BRAF*) may be found in *MYD88* wild-type tumors [71,75]. Similarly, recurrent alterations in tumor suppressor genes, such as *CDKN2A*, *HLA*, *B2M*, *CIITA*, and *CD58*, can impair antigen presentation and T-cell co-stimulation, which contribute to immune evasion [71,75]. These alterations could interfere with tumor surveillance and permit the emergence of additional malignancies. In other CBCL subtypes, chromatin-modifying genes, such as *CREBBP*, *KMT2D*, and *EZH2*, are involved in transcriptional regulation and DNA repair, both of which are crucial for maintaining genomic integrity and preventing oncogenic mutations [76,77].

Another potential contributor is chronic antigenic stimulation, a mechanism long hypothesized in CBCL. Previous studies have revealed a close relationship between *Borrelia burgdorferi* and cutaneous lymphoma, although this was limited to endemic areas [78]. Other infectious agents, such as the Epstein–Barr virus and human herpes virus, have also been reported [79]. One case report documented two cases of CBCL progressing from actinic prurigo [80]. Based on the concept that CBCL could be derived from reactive processes driven by antigen exposure, the author proposed a possible correlation between UV radiation and CBCL [80]. To some extent, autoimmune diseases, such as Sjögren’s syndrome and autoimmune thyroid disease, may be associated with CBCL, making autoantigens another probable source for constant stimulation [81]. This continuous activation promotes the recruitment of kinases such as SYK and BTK, leading to NF-κB pathway activation, further promoting the transcription of the genes that inhibit apoptosis and support cell proliferation [82,83]. Notably, NF-κB activation is not exclusive to lymphoid malignancies; it has also been observed in various solid tumors, like breast, lung, and prostate cancers [84,85,86,87].

In short, genetic susceptibility and chronic antigenic stimulation may contribute to CBCL oncogenesis. However, these propositions remain speculative and are supported by limited evidence. A deeper understanding of the biological interplay between genetic abnormalities, chronic antigen exposure, and immune dysregulation is essential to elucidate these mechanisms.

## 6. Conclusions

In light of the findings from all the studies above, integrating vigilant cancer surveillance, patient education, and constant research efforts is essential to guide evidence-based management and follow-up plans for patients with cutaneous lymphoma. Moreover, physicians should raise awareness and proactively monitor SPM development in these patients. For CTCL, especially MF, Goyal et al. had proposed comprehensive screening strategies for NHL, HL, lung cancer, bladder cancer, and melanoma, the five most-probable SPM based on meta-analysis performed by the same research group [20,21]. As for CBCL, more compelling evidence is required. So far, SEER-based studies have highlighted the high risks of hematologic malignancies, lung, renal, thyroid, bladder, prostate cancers and melanomas [47,48]. Although widely accepted screening guidelines for most of the malignancies listed above are lacking, the US Preventive Services Task Force and Taiwan Ministry of Health and Welfare provide recommendations for cancer screening, including lung and breast cancers. For skin cancers such as melanoma and Merkel cell carcinoma, full body skin examination can be swiftly performed at a dermatologic outpatient clinic. To compensate for the gap in current guidelines, patients should be educated on common cancer-related symptoms, such as unintentional weight loss, night sweats, hoarseness, hemoptysis, hematuria, new pigmented skin lesions, and breast lumps.

Future research should prioritize large-scale prospective cohort studies to better define the true incidence, timing, and risk factors associated with SPM development in cutaneous lymphoma. The PROCLIPI international registry can serve as a useful tool to assess prognosis of CTCL [88]. Given the observed discrepancies between the Western and Asian populations, and scarcity of studies focusing on specific races, research regarding geographic differences are warranted. Another discrepancy lies in the definition of latency from the initial PCL diagnosis. To what extent should another cancer be regarded as a new primary tumor rather than a concurrent tumor? The indolent course and diagnostic difficulties of PCL also made their true onset ambiguous. Finally, elucidating genetic and molecular mechanisms is essential. Future investigations should put more effort into comprehensive molecular profiling and the integration of genomic, transcriptomic, and epigenetic analyses of patients with PCLs who develop SPMs. Such approaches are crucial for risk stratification, refining personalized surveillance strategies, and reducing the long-term cancer burden in this vulnerable patient population.

## Figures and Tables

**Table 1:** Selected studies focusing on second primary malignancies after mycosis fungoides and Sézary syndrome.

Reference	Subtype	Database (Study Period)	No. of Patients	Statistical Significance	CTCL to SPM, Median Time	Predominant SPM, n (RR)
Kantor et al. [11]	MF & SS	SEER program (1973–1983)	544	35 pts (6.4%), RR = 1.74	NR	Colon cancer, 7 (RR, 3.1) Lung cancer, 10 (RR, 2.8) NHL, 3 (RR, 5.5)
Huang et al. [12]	MF & SS	SEER program (1984–2001)	1798	197 pts (11.0%), RR = 1.32 (95% CI, 1.15–1.52)	49 months	HL, 6 (SIR, 17.14 [95% CI, 6.25–37.26]) NHL, 27 (SIR, 5.08 [95% CI, 3.34–7.38]) Melanoma, 10 (SIR, 2.60 [95% CI, 1.25–4.79]) Urinary cancer, 21 (SIR, 1.74 [95% CI, 1.08–2.66])
Goyal et al. [13]	MF	SEER program (2000–2015)	6742	511 pts (7.6%), SIR = 10.15 (95% CI, 9.29–11.07)	3 years (0–15)	HL, 12 (SIR, 100.14 [95% CI, 49.99–179.19]) NHL, 140 (SIR, 30.48 [95% CI, 22.06–41.06]) Colorectal cancer, 36 (SIR, 6.99 [95% CI, 4.90–9.68]) Pancreatic cancer, 10 (SIR, 6.84 [95% CI, 3.28–12.57] Lung and bronchus cancer, 65 (SIR, 8.34 [95% CI, 6.44–10.63]) Melanoma, 20 (SIR, 9.00 [95% CI, 5.50–13.90]) Female breast cancer, 47 (SIR, 11.28 [95% CI, 8.29–15.00]) Prostate cancer, 2 (SIR, 5.65 [95% CI, 4.30–7.28]) Bladder cancer, 59 (SIR, 4.35 [95% CI, 2.38–7.30]) Renal cancer, 14 (SIR, 3.62 [95% CI, 1.33–7.87])
Almukhtar et al. [14]	MF & SS	SEER program (1994–2014)	4229	550 pts (13.0%), SIR = 1.26 (95% CI, 1.16–1.37)	NR	Lung cancer, 82 (SIR, 1.33 [95% CI, 1.05–1.65]) Chronic lymphocytic leukemia, 14 (SIR, 2.5 [95% CI, 1.41–4.32]) HL, 13 (SIR, 11.18 [95% CI, 5.95–19.12]) NHL, 98 (SIR, 5.28 [95% CI, 4.29–6.44]).
Ai et al. [15]	MF & SS *	SEER program (1973–2009)	195	9 pts (4.6%), SIR = 3.40 (95% CI, 1.55–6.45)	9 years (1–28)	Lymphoma, <5 (SIR, 12.86 [95% CI, 2.65–37.59]) Melanoma, <5 (SIR, 9.31 [95% CI, 8.75–33.62]).
Väkevä et al. [16]	MF & SS	Finnish Cancer Registry (1953–1995)	319	36 cancers, SIR = 1.4 (95% CI, 1.0–1.9)	NR	Lung cancer, 12 (SIR, 2.7 [95% CI, 1.4–4.8]) Lymphomas, 2 (SIR, 7.0 [95% CI, 1.9–18])
Lindahl et al. [17]	MF	Danish Cancer Registry (1979–2008)	386	65 pts (16.9%), SIR = 1.2 (95% CI, 0.9–1.5)	NR	NHL, 8 (SIR, 5.2 [95% CI, 2.4–9.8])
Ai et al. [15]	MF & SS *	California Cancer Registry (1988–2009)	204	<5 pts, SIR = 3.45 (95% CI, 0.94–8.83)§	NR	Melanoma, <5 (SIR, 6.88 [95% CI, 0.17–38.32]) ^†^
Huang et al. [12]	MF & SS	Stanford (1973–2001)	429	37 pts (8.6%), RR = 1.04 (95% CI, 0.76–1.44)	4 years	HL, 3 (SIR, 27.27 [95% CI, 5.35–77.54]) Biliary cancer, 2 (SIR, 11.76 [95% CI, 1.51–42.02])
Brownell et al. [18]	MF	MD Anderson Cancer Center (1979–1999)	672	37 pts (5.5%), SIR = 1.79 (95% CI, 1.22–2.39)	25 months (3–138)	NHL, 7 (SIR, 9.87 [95% CI, 3.96–20.31]) HL, 2 (SIR, 25.56 [95% CI, 3.03–90.31]) Acute myeloid leukemia, 3 (SIR, 22.39 [95% CI, 4.76–67.44]) Vulvar cancer, 2 (SIR, 29.86 [95% CI, 3.46–103.21])

* The study only included patients under 30 years old ^†^. Abbreviation: The risk was increased but not statistically significant CI, confidence interval; CTCL, cutaneous T-cell lymphoma; HL, Hodgkin lymphoma; MF, mycosis fungoides; NHL, non-Hodgkin lymphoma; No, number; NR, not recorded; “pts, patients; RR, relative risk; SEER, Surveillance, Epidemiology, and End Results; SIR, standardized incidence ratio; SPM, second primary malignancy; SS, Sézary syndrome.

**Table 2:** Selected studies focusing on second primary malignancies after primary cutaneous CD30-positive T-cell lymphoproliferative disorders *.

Reference	Subtype	Database (Study Period)	No. of Patients	Statistical Significance	CTCL to SPM, Median Time	Predominant SPM, n (RR)
Melchers et al. [29]	LyP	Multicenter (1985–2018)	504	HM: 39 in 465 pts (8.4%), RR = 11.9 (95% CI, 8.3–15.5)	68 months (3–286)	MF, 11; ALCL, 20
				non-HM: NR, RR = 2.8 (95% CI, 2.4–3.3)	NR	Cutaneous squamous cell carcinoma, 26(RR, 4.3 [95% CI, 2.5–6.1]) Melanoma, 15 (RR, 4.2 [95% CI, 1.8–6.6]) Lung cancer, 21 (RR, 3.7 [95% CI, 2.1–5.2]) Intestinal & rectal cancer, 13 (RR, 2.4 [95% CI, 1.2–3.7]) Bladder cancer, 11 (RR = 8.1, 95% CI, 3.1–13.1)
Cordel et al. [30]	LyP	Multicenter (1991–2006)	106	17 pts (16.0%) had 21 SPMs	5 years (1.5–7)	MF, 8; ALCL, 6
de Souza et al. [31]	LyP	Mayo Clinic (1991–2008)	123	8 pts (6.5%) had 9 SPMs	NR	-
Wieser et al. [32]	LyP	MD Anderson Cancer Center (1999–2015)	180	47 pts (26.1%)	NR	MF, 30
Amber et al. [22]	pc-ALCL	SEER program (1992–2011)	NR	NR, SIR = 1.63 (95% CI, 1.07–2.39)	NR	HL, NR (SIR, 39.99 [95% CI, 4.84–144.47]) NHL, NR (SIR, 5.84 [95% CI, 1.59–14.95]) Urinary system cancers, NR (SIR, 3.22 [95% CI, 1.04–7.51])
Joshi et al. [33]	pc-ALCL	SEER program (1973–2020)	569	NR, SIR = 1.53 (95% CI, 1.19–1.94)	NR	HL, 2 (SIR, 16.35 [95% CI, 1.98–59.06]) NHL, 11 (SIR, 5.67 [95% CI, 2.83–10.15]) Melanoma, 10 (SIR, 3.91 [95% CI, 1.88–7.19]) Kidney cancers, 5 (SIR, 3.64 [95% CI, 1.18–8.50]) Respiratory system cancers, 12 (SIR, 2.00 [95% CI, 1.03–3.49])

* Only SPM that developed after initial diagnosis of lymphomas are included. Abbreviation: CTCL, cutaneous T-cell lymphoma; HM, hematologic malignancy; HL, Hodgkin lymphoma; LyP, lymphomatoid papulosis; MF, mycosis fungoides; NHL, non-Hodgkin lymphoma; No, number; NR, not recorded; pc-ALCL, primary cutaneous anaplastic large cell lymphoma; pts, patients; RR, relative risk; SEER, Surveillance, Epidemiology, and End Results; SIR, standardized incidence ratio; SPM, second primary malignancy.

**Table 3:** Selected studies focusing on second primary malignancies associated with primary cutaneous B cell lymphoma.

Reference	Database (Study Period)	No. of Patients	Statistical Significance	C B CL to SPM, Median Time	Predominant SPM, n (RR)	Most Risky Subtype
Banner et al. [46]	SEER program (2000–2019)	5179	NR for all SPMs	CM: 1~5 years	CM, 36 (SIR, 1.35 [95% CI, 0.94–1.86])	PCFCL
				MCC: <1 year	MCC, 3 (SIR, 3.74 [95% CI, 0.77–10.92])	NR
Banner et al. [47]	SEER program (2000–2020)	5435	847 pts (15.6%), SIR = 1.54 (95% CI, 1.43–1.64)	<1 year	Thyroid, 17 (SIR, 2.27 [95% CI, 1.32–3.63]) Renal, 30 (SIR, 1.54 [95% CI, 1.04–2.20]) Melanoma, 40 (SIR, 1.35 [95% CI, 0.96–1.83]) Lung, 93 (SIR, 1.22 [95% CI, 0.98–1.49]) Bladder, 40 (SIR, 1.21 [95% CI, 0.87–1.65]) Prostate, 118 (SIR, 1.29 [95% CI, 1.07–1.54])	NR
Shah et al. [48]	SEER program (2000–2020)	3757	343 pts (9.1%), SIR = 1.37 (95% CI, 1.23–1)	11.09 years	Lymphoma, 93 (SIR, 8.01 [95% CI, 6.46–9.81]) Leukemia, 13 (SIR, 1.72 [95% CI, 0.91–2.94]) Prostate cancer, 59 (SIR, 1.49 [95%CI 1.13–1.92]) Male genital cancer, 59 (SIR, 1.46 [95% CI, 1.11–1.88]) Skin tumor (excluding BCC and SCC), 17 (SIR, 1.16 [95% CI, 0.67–1.85])	NR
Avallone et al. [43]	Multicenter	144	40 pts (27.8%) *	48 months	BCC, 19 ^†^	PCFCL
Chan et al. [44]	Birmingham Specialist Cutaneous Lymphoma Service	51	13 pts (25.5%) *	NR	BCC, 6 *	NR
Gómez Sánchez et al. [45]	Hospital General de Villarrobledo (1993–2014)	36	6 pts (16.7%) *	0.43 years	-	PCMZL

* In this study, the second primary malignancies include all previous, synchronous, and metachronous cancers. ^†^ Only synchronous and metachronous BCC are included. Abbreviation: BCC, basal cell carcinoma; CBCL, cutaneous B-cell lymphoma; CM, cutaneous melanoma; MCC, Merkel cell carcinoma; No, number; NR, not recorded; PCFCL, primary cutaneous follicle center lymphoma; PCMZL, primary cutaneous marginal zone lymphoma; SCC, squamous cell carcinoma; SEER, Surveillance, Epidemiology, and End Results; SIR, standardized incidence ratio; SPM, second primary malignancy.

## Data Availability

No new data were created or analyzed in this study. Data sharing is not applicable to this article.

## Supplementary Materials

The following supporting information can be downloaded at: https://www.mdpi.com/article/10.3390/diagnostics15243150/s1. Table S1: Immunohistochemical profile and differential diagnoses of primary cutaneous lymphomas.

## Abbreviations

The following abbreviations are used in this manuscript:

ALCL	anaplastic large cell lymphoma
BCC	basal cell carcinoma
CAF	cancer-associated fibroblast
CBCL	cutaneous B-cell lymphoma
CI	confidence interval
CTCL	cutaneous T-cell lymphoma
EBV	Epstein–Barr virus
EORTC	European Organization for Research and Treatment of Cancer
HL	Hodgkin lymphoma
HR	hazard ratio
LyP	lymphomatoid papulosis
MF	mycosis fungoides
NHL	non-Hodgkin lymphoma
OR	odds ratio
Pc-ALCL	primary cutaneous anaplastic large cell lymphoma
PCFCL	primary cutaneous follicle center lymphoma
PCMZL	primary cutaneous marginal zone lymphoma
PCDLBCL-LT	primary cutaneous diffuse large B-cell lymphoma, leg type
PCL	primary cutaneous lymphoma
SCC	squamous cell carcinoma
SEER	Surveillance, Epidemiology, and End Results
SIR	standardized incidence ratio
SPM	second primary malignancy
SS	Sézary syndrome
UV	ultraviolet
