# Supplementary material for: Association of Secondary Primary Malignancies in Cutaneous Lymphoma: A Narrative Review
Source: Diagnostics (Basel). 2025 Dec 11;15(24):3150. doi: 10.3390/diagnostics15243150 (PMC12731868; doi:10.3390/diagnostics15243150)
Supplement: Supplementary file 1 [file diagnostics-15-03150-s001.zip › diagnostics-4001581-supplementary.pdf]

Supplementary Table S1. Immunohistochemical profile and differential diagnoses of primary cutaneous lymphomas.

Cutaneous Lymphoma	Immunohistochemical Profile	Differential Diagnoses
MF/SS	CD3^{+}, CD4^{+}, CD5^{+}, CD8^{-}, CD45RO^{+}, often loss of CD7, TIA-1^{-}	LyP (type B)<br>CD8^{+} AECTCL<br>Gamma/delta T-cell lymphoma
LyP	CD3^{+}, variable CD4/CD8, CD30^{+} (strong, >75%)	PC ALCL<br>Infectious skin diseases<br>MF
PC ALCL	CD3^{+}, CD4^{+}, CD30^{+} (diffuse, >75%), ALK^{-}, EMA^{+}	LyP<br>Systemic ALCL<br>CD30^{+} MF
SPTCL	CD3^{+}, CD4^{-}, CD8^{+}, cytotoxic markers (TIA-1, granzyme B, perforin), βF1^{+}, CD56^{-}	Lupus panniculitis<br>Gamma/delta T-cell lymphoma<br>Extranodal NK/T-cell lymphoma
PC gamma/delta T-cell lymphoma	CD3^{+}, CD4^{-}, variable CD8, cytotoxic markers, TCRγδ^{+}, CD56^{+}, βF1^{-}	SPTCL<br>Extranodal NK/T-cell lymphoma
CD8^{+} AECTCL	CD3^{+}, CD4^{-}, CD8, CD45RO^{+}, CD45RO^{-}, cytotoxic markers, βF1^{+}	LyP (type D)<br>MF (CD8^{+} variant)<br>Viral exanthem
CD4^{+} SMTLPD	CD3^{+}, CD4^{+}, CD7^{-}, CD8^{-}, CD30^{-}, follicular helper markers (PD-1, ICOS, BCL6)	SPTCL<br>MF (tumor stage)<br>Pseudolymphoma
PTCL, NOS	CD3^{+}, variable CD4/CD8, variable cytotoxic markers, often loss of pan-T markers	MF<br>CD8^{+} AECTCL
PCMZL	CD20^{+}, CD79a^{+}, CD5^{-}, CD10^{-}, CD23^{-}, BCL2^{+}, BCL6^{-}, light chain restriction, cyclin-D1^{-}	Pseudolymphoma<br>PCFCL
PCFCL	CD20^{+}, CD79a^{+}, CD5^{-}, variable CD10, CD43^{-}, BCL2^{-}, BCL6^{+}, MUM-1^{-}	Pseudolymphoma<br>PCMZL
PCDLBCL, LT	CD20^{+}, CD79a^{+}, CD5^{-}, CD10^{-}, BCL2^{+}, BCL6^{+}, MUM1^{+}, high Ki-67	PCFCL<br>PCMZL
EBV^{+} MCU	Variable CD20 and CD79, CD10^{-}, CD30^{+}, BCL6^{-},EBV-encoded RNA (EBER)$^{+}$, PAX5^{+}	EBV^{+}PCDLBCL<br>EBV^{+}Hodgkin lymphoma<br>Lymphomatoid granulomatosis

* ALCL, anaplastic large cell lymphoma; CD4^{+} SMTLPD, CD4^{+} small-medium T-cell lymphoproliferative disorder; CD8^{+} AECTCL, CD8^{+} aggressive epidermotropic cytotoxic T-cell lymphoma; EBV, Epstein-Barr virus; LyP, lymphomatoid papulosis; MCU, mucocutaneous ulcer; MF/SS, mycosis fungoides / Sézary syndrome; NK, natural killer; PC, primary cutaneous; PCMZL, primary cutaneous marginal zone lymphoma; PCDLBCL, LT, primary cutaneous diffuse large B-cell lymphoma, leg type; PCFCL, primary cutaneous follicle center lymphoma; PTCL, NOS, peripheral T-cell lymphoma, not otherwise specified; SPTCL, subcutaneous panniculitis-like T-cell lymphoma
